# Supplementary material for: Incidence and risk of post-COVID-19 thromboembolic disease and the impact of aspirin prescription; nationwide observational cohort at the US Department of Veteran Affairs
Source: PLoS One. 2024 Sep 17;19(9):e0302612. doi: 10.1371/journal.pone.0302612 (PMC11407644; doi:10.1371/journal.pone.0302612)

**Supplementary 2 Figure.** Density Plots Displaying the Balance on the Matched Covariates of Age, Gender, and the VA’s Care Assessment of Needs (CAN) 1-Year Mortality Score Before and After Propensity Score Matching using the “nearest neighbor” method in R Studio (Version 3.6.2).


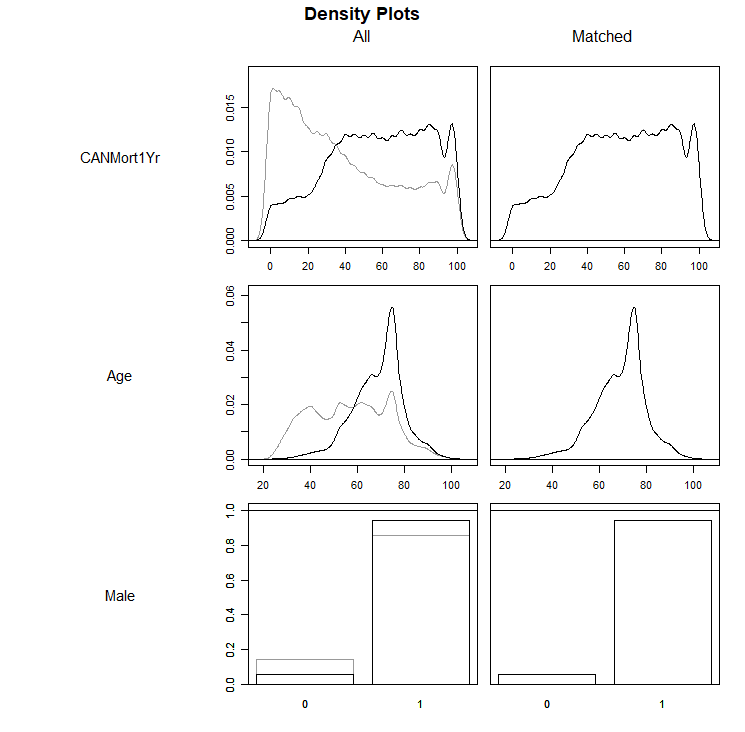

Supplement: S1 Fig — (DOCX) [file pone.0302612.s001.docx]
