# Supplementary material for: Incidence and risk of post-COVID-19 thromboembolic disease and the impact of aspirin prescription; nationwide observational cohort at the US Department of Veteran Affairs
Source: PLoS One. 2024 Sep 17;19(9):e0302612. doi: 10.1371/journal.pone.0302612 (PMC11407644; doi:10.1371/journal.pone.0302612)
Supplement: S2 Fig — (DOCX) [file pone.0302612.s002.docx]

**Supplemental 4 Figure.** Subgroup analyses of the odds of post-COVID-19 acute thromboembolic disorders using the complete cohort (N=334,374).

**
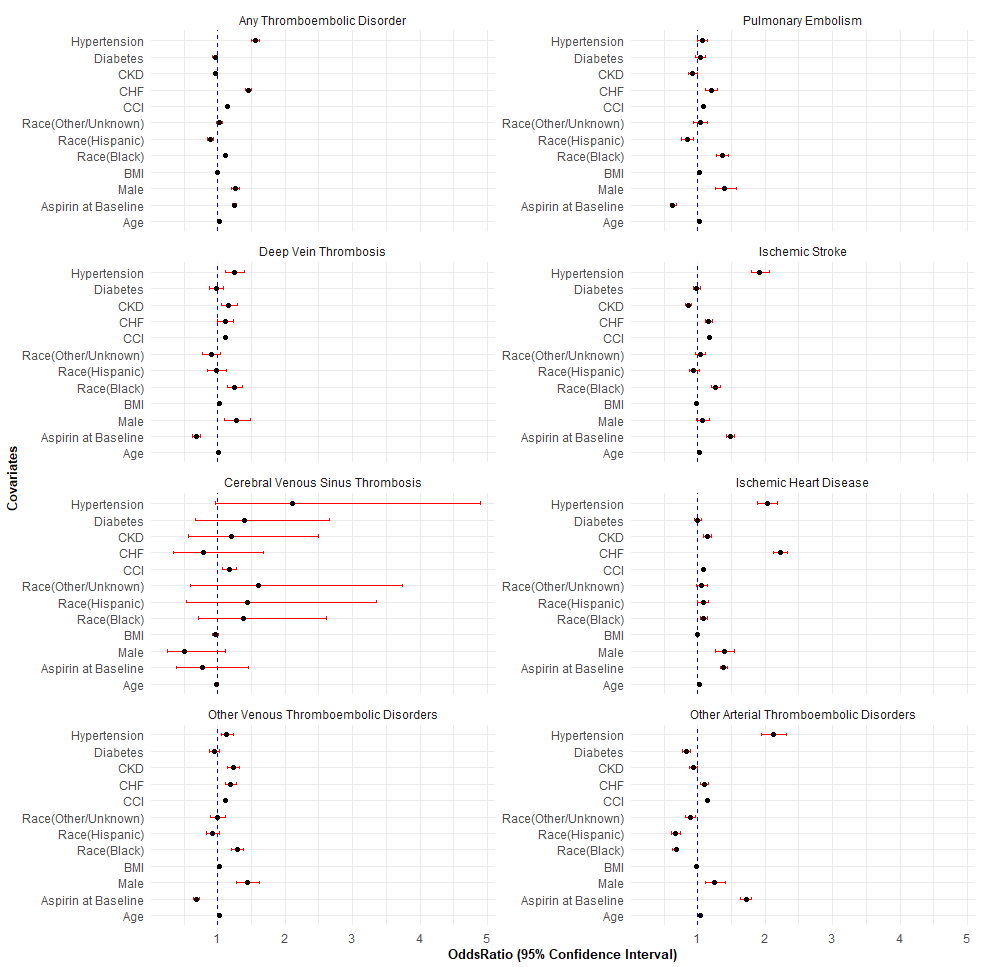
**
