# Supplementary material for: Incidence and risk of post-COVID-19 thromboembolic disease and the impact of aspirin prescription; nationwide observational cohort at the US Department of Veteran Affairs
Source: PLoS One. 2024 Sep 17;19(9):e0302612. doi: 10.1371/journal.pone.0302612 (PMC11407644; doi:10.1371/journal.pone.0302612)
Supplement: S1 Table — (DOCX) [file pone.0302612.s003.docx]

**Supplementary 1 Table:** ICD-10 Codes and Definitions for Acute Thromboembolic Disorders Included in the Analysis

| **Pulmonary embolism****^[[1]](#footnote-1)^** | |
| --- | --- |
| I26.% | Pulmonary embolism |
| **Deep vein thrombosis^[[2]](#footnote-2),^^[[3]](#footnote-3) ,^^[[4]](#footnote-4)^** | |
| I80.1% | Phlebitis and thrombophlebitis of femoral vein |
| I80.2% | Phlebitis and thrombophlebitis of other and unspecified deep vessels of lower extremities |
| I80.3 | Phlebitis and thrombophlebitis of lower extremities, unspecified |
| I80.8-9 | Phlebitis and thrombophlebitis of unspecified site |
| I82.8% | Embolism and thrombosis of other specified veins |
| I82.90 | Acute embolism and thrombosis of unspecified vein |
| **Ischemic Stroke** | |
| I63.% | Cerebral infarction |
| G45.% | Transient cerebral ischemic attacks and related syndromes |
| **Cerebral Venous Sinus Thrombosis^[[5]](#footnote-5)^** | |
| I63.6 | Cerebral infarction due to cerebral venous thrombosis, nonpyogenic |
| G08 | Intracranial and intraspinal phlebitis and thrombophlebitis |
| I67.6 | Nonpyogenic thrombosis of intracranial venous system - *aka cavernous sinus syndrome* |
| **Acute Ischemic Heart Disease^[[6]](#footnote-6)^** | |
| I21.% | Acute myocardial infarction (including STEMI/NSTEMI) |
| I22.% | Subsequent ST elevation (STEMI) and non-ST elevation (NSTEMI) myocardial infarction |
| I23.% | Complications following acute STEMI/NSTEMI |
| I24.% | Acute coronary thrombosis not resulting in myocardial infarction |
| **Other acute venous thrombosis and embolism** | |
| I80.0% | Phlebitis and thrombophlebitis of superficial vessels of lower extremities |
| I82.A1% | Acute embolism and thrombosis of axillary vein |
| I82.B1% | Acute embolism and thrombosis of subclavian vein |
| I82.C1% | Acute embolism and thrombosis of internal jugular vein |
| I82.210 | Acute Embolism and thrombosis of superior vena cava |
| I82.220 | Acute embolism and thrombosis of inferior vena cava |
| I82.290 | Acute embolism and thrombosis of other thoracic veins |
| I82.3 | Embolism and thrombosis of renal vein |
| I82.4% | Acute embolism and thrombosis of other specified deep veins of lower extremity |
| I82.6% | Acute embolism and thrombosis of veins of upper extremity |
| **Other acute arterial thrombosis and embolism^[[7]](#footnote-7)^** | |
| I74.% | Arterial embolism and thrombosis |
| H34.% | Retinal embolism and thrombosis |
| *I62.9 was included as could identify possible cases of CVST because some might present with intracerebral hemorrhage, according to prior study: Meng-Tsang Hsieh et al. (2021) Validation of ICD-10-CM Diagnosis Codes for Identification of Patients with Acute Hemorrhagic Stroke in a National Health Insurance Claims Database, Clinical Epidemiology, 13:, 43-51, DOI: [10.2147/CLEP.S288518](https://doi.org/10.2147/CLEP.S288518) | |

1. Burles, K., Innes, G., Senior, K. et al. Limitations of pulmonary embolism ICD-10 codes in emergency department administrative data: let the buyer beware. BMC Med Res Methodol 17, 89 (2017). https://doi.org/10.1186/s12874-017-0361-1 [↑](#footnote-ref-1)
2. US Department of Veterans Affairs . Corporate data warehouse (CDW). URL: [www.hsrd.research.va.gov/for_researchers/vinci/cdw.cfm](http://www.hsrd.research.va.gov/for_researchers/vinci/cdw.cfm). [↑](#footnote-ref-2)
3. Verma AA, Masoom H, Pou-Prom C, Shin S, Guerzhoy M, Fralick M, Mamdani M, Razak F. Developing and validating natural language processing algorithms for radiology reports compared to ICD-10 codes for identifying venous thromboembolism in hospitalized medical patients. Thromb Res. 2022 Jan;209:51-58. doi: 10.1016/j.thromres.2021.11.020. Epub 2021 Nov 27. PMID: 34871982. [↑](#footnote-ref-3)
4. Boulay F, Berthier F, Schoukroun G, Raybaut C, Gendreike Y, Blaive B. Seasonal variations in hospital admission for deep vein thrombosis and pulmonary embolism: analysis of discharge data. BMJ. 2001 Sep 15;323(7313):601-2. doi: 10.1136/bmj.323.7313.601. PMID: 11557707; PMCID: PMC55575. [↑](#footnote-ref-4)
5. Liao SC, Shao SC, Lai EC, Lin SJ, Huang WI, Hsieh CY. Positive Predictive Value of ICD-10 Codes for Cerebral Venous Sinus Thrombosis in Taiwan's National Health Insurance Claims Database. Clin Epidemiol. 2022 Jan 3;14:1-7. doi: 10.2147/CLEP.S335517. PMID: 35018122; PMCID: PMC8740620. [↑](#footnote-ref-5)
6. Burles, K., Innes, G., Senior, K. et al. Limitations of pulmonary embolism ICD-10 codes in emergency department administrative data: let the buyer beware. BMC Med Res Methodol 17, 89 (2017). https://doi.org/10.1186/s12874-017-0361-1 [↑](#footnote-ref-6)
7. American Academy of Professional Coders (AAPC) List of Codes for arterial embolism and thrombosis: [ICD-10 Code for Arterial embolism and thrombosis- I74- Codify by AAPC](https://www.aapc.com/codes/icd-10-codes/I74#:~:text=ICD%2D10%20code%20I74%20for,Diseases%20of%20the%20circulatory%20system%20.) [↑](#footnote-ref-7)
