# Supplementary material for: Incidence and risk of post-COVID-19 thromboembolic disease and the impact of aspirin prescription; nationwide observational cohort at the US Department of Veteran Affairs
Source: PLoS One. 2024 Sep 17;19(9):e0302612. doi: 10.1371/journal.pone.0302612 (PMC11407644; doi:10.1371/journal.pone.0302612)
Supplement: S2 Table — (DOCX) [file pone.0302612.s004.docx]

**Supplementary 3 Table.** Sub-analysis cohort characteristics among those with and without thromboembolic disorders within 12 months following the patient's index date using the complete cohort (N=334,374).

|  | No acute thrombotic events post COVID-19 (N=292,920) | Any acute thrombotic events post COVID-19  (N=41,454) |
| --- | --- | --- |
| Age (years), mean (SD) | 58.5 (16.1) | 70.6 (11.9) |
| Male | 255053 (87.1%) | 39288 (94.8%) |
| Race and/or Ethnicity |  |  |
| White | 163996 (56.0%) | 24595 (59.3%) |
| Black or African American | 66998 (22.9%) | 10409 (25.1%) |
| Hispanic | 31764 (10.8%) | 3064 (7.4%) |
| Asian | 4076 (1.4%) | 246 (0.6%) |
| American Indian or Alaska Native | 2983 (1.0%) | 406 (1.0%) |
| Native Hawaiian/Other Pacific Islander | 2581 (0.9%) | 314 (0.8%) |
| Unknown | 12820 (4.4%) | 1856 (4.5%) |
| Missing | 7702 (2.6%) | 564 (1.4%) |
| CAN 1 Year Mortality Score, mean (SD) | 40.0 (29.5) | 66.8 (27.1) |
| Prescribed Aspirin prior to COVID-19 Dx | 225827 (77.1%) | 24542 (59.2%) |
| Aspirin Dosage (mg/day), mean (SD) | 96.5 (58.7) | 106 (73.0) |
| None | 226031 (77.2%) | 24338 (58.7) |
| 81 mg/day | 62071 (21.2%) | 14905 (36.0%) |
| 162 mg/day | 843 (0.3%) | 329 (0.8%) |
| 243 mg/day | 37 (0.0%) | 13 (0.0%) |
| 325 mg/day | 3852 (1.3%) | 1576 (3.8%) |
| 405-650 mg/day | 31 (0.0%) | 12 (0.0%) |
| No Dose Listed (Non-VA prescription) | 449 (0.3%) | 116 (0.4%) |
| CCI Score, mean (SD) | 2.34 (2.83) | 5.21 (3.60) |
| Body Mass Index (BMI), mean (SD) | 30.9 (6.37) | 29.8 (6.71) |
| Common Comorbidities Prior to COVID-19 Dx |  |  |
| Congestive Heart Failure | 32776 (11.2%) | 14131 (34.1%) |
| Chronic Pulmonary Disease | 99190 (33.9%) | 21842 (52.7%) |
| Chronic Kidney Disease | 41334 (14.0%) | 14829 (34.6%) |
| Diabetes | 42471 (14.5%) | 6904 (16.7%) |
| Hypertension | 170203 (58.1%) | 35591 (85.9%) |
| Inpatient for COVID-19 | 74447 (25.4%) | 28848 (69.6%) |
| COVID-19 Vaccinated prior to COVID-19 Dx | 92917 (31.7%) | 13036 (31.4%) |
| Number Vaccine Doses prior to COVID-19 Dx |  |  |
| Unvaccinated | 200003 (68.3%) | 28418 (68.6%) |
| 1 Dose | 9879 (3.4%) | 1134 (2.7%) |
| 2 Doses | 43411 (14.8%) | 5356 (12.9%) |
| 3 Doses | 39627 (13.5%) | 6546 (15.8%) |
| Death within follow-up period | 19051 (6.5%) | 8955 (21.6%) |
